# Genetically Engineered Extracellular Vesicles Harboring Transmembrane Scaffolds Exhibit Differences in Their Size, Expression Levels of Specific Surface Markers and Cell-Uptake

**DOI:** 10.3390/pharmaceutics14122564

**Published:** 2022-11-23

**Authors:** Jiayi Zhang, Annie Brown, Brendan Johnson, David Diebold, Kyle Asano, Gerard Marriott, Biao Lu

**Affiliations:** 1 Department of Bioengineering, School of Engineering, Santa Clara University, 500 El Camino Real, Santa Clara, CA 95053, USA; 2 Department of Bioengineering, University of California at Berkeley, Berkeley, CA 94720, USA

**Keywords:** extracellular vesicles (EVs), exosome, CD9, CD63, CD81, vesicular stomatitis virus envelope glycoprotein (VSVG), nanotechnology, targeted drug delivery

## Abstract

Background: Human cell-secreted extracellular vesicles (EVs) are versatile nanomaterials suitable for disease-targeted drug delivery and therapy. Native EVs, however, usually do not interact specifically with target cells or harbor therapeutic drugs, which limits their potential for clinical applications. These functions can be introduced to EVs by genetic manipulation of membrane protein scaffolds, although the efficiency of these manipulations and the impacts they have on the properties of EVs are for the most part unknown. In this study, we quantify the effects of genetic manipulations of different membrane scaffolds on the physicochemical properties, molecular profiles, and cell uptake of the EVs. Methods: Using a combination of gene fusion, molecular imaging, and immuno-based on-chip analysis, we examined the effects of various protein scaffolds, including endogenous tetraspanins (CD9, CD63, and CD81) and exogenous vesicular stomatitis virus glycoprotein (VSVG), on the efficiency of integration in EV membranes, the physicochemical properties of EVs, and EV uptake by recipient cells. Results: Fluorescence imaging and live cell monitoring showed each scaffold type was integrated into EVs either in membranes of the endocytic compartment, the plasma membrane, or both. Analysis of vesicle size revealed that the incorporation of each scaffold increased the average diameter of vesicles compared to unmodified EVs. Molecular profiling of surface markers in engineered EVs using on-chip assays showed the CD63-GFP scaffold decreased expression of CD81 on the membrane surface compared to control EVs, whereas its expression was mostly unchanged in EVs bearing CD9-, CD81-, or VSVG-GFP. The results from cell uptake studies demonstrated that VSVG-engineered EVs were taken up by recipient cells to a greater degree than control EVs. Conclusion: We found that the incorporation of different molecular scaffolds in EVs altered their physicochemical properties, surface protein profiles, and cell-uptake functions. Scaffold-induced changes in the physical and functional properties of engineered EVs should therefore be considered in engineering EVs for the targeted delivery and uptake of therapeutics to diseased cells.

## 1. Introduction

Extracellular vesicles (EVs) are cell-derived nanocarriers that are thought to mediate cell-to-cell communication in prokaryotes and eukaryotes [1,2]. In mammals, EVs are continuously synthesized and released to the extracellular environment in a constitutive and regulated manner [3,4]. Mammalian EVs can be divided into two distinct types according to their membrane origin: for example, microvesicles have a diameter of 100–1000 nm and are formed by direct budding from the plasma membrane, while exosomes (50–150 nm) are released via exocytosis of multivesicular bodies (MVBs) [5,6]. EVs can also be divided into CD9^+^, CD63^+^, and CD81^+^ subtypes, each bearing specific surface markers [7,8,9]. After being released from host cells, EVs may be found in significant numbers in blood plasma [10], lymph [11], ammonic fluid [12], saliva [13], urine [14], breast milk [15], and seminal fluid [16]. Consequently, they may be involved in shuttling specific signaling molecules, including proteins, nucleic acids, and lipids, to target cells in remote tissues [17,18]. EVs are known to regulate tissue growth and organ development [19,20], modulate the immune response [21,22,23], mediate inflammation and viral infection [24,25,26], and promote cancer progression and metastasis [27,28,29]. As a new generation of nanomaterials, EVs may offer advantages over artificial nano-vehicles such as liposomes and polymers for targeted drug delivery and therapy [30,31]. First, EVs are derived from human cells, so they are biocompatible and biodegradable and may evade rapid clearance by the immune system [32]. Second, EVs are capable of penetrating tissue to reach deep-seated cells, and there are known cases where they cross the blood-brain barrier (BBB) [33]. Third, endogenously and/or exogenously produced EVs can be genetically engineered to facilitate drug loading and targeted delivery of biologics to diseased tissue [34,35].

While EVs hold great promise in nanomedicine, endogenous EVs exhibit poor targeting to diseased cells and lack therapeutic cargo [36,37]. These functions, however, can be introduced through the use of molecular and genetic engineering techniques [38]. An essential requirement here is to identify molecular scaffolds that present a tissue-targeting moiety while enabling loading with a therapeutic agent. To this end, previous studies have examined the use of membrane-associated proteins, including CD9, CD63, and CD81, to display tissue-targeting or imaging moieties on the outer surface of exosomes [39,40]. In addition, we have reported that the viral envelope glycoprotein derived from the vesicular stomatitis virus glycoprotein (VSVG) is a suitable scaffold for loading bioactive enzymes associated with lysosomal storage disorders in the lumen of the exosome [41,42]. Moreover, El-Addloussi et al. used the lysosome-associated membrane protein 2B (LAMP2B) to install neurotrophic peptides in the lumen of exosomes for targeted delivery of siRNA cargo to the brain in a mouse model [43]. Lipid-anchoring proteins/peptides may be used as an alternative membrane scaffold to present disease-targeting agents on the outer surface of EVs. For example, the C1C2 domain of lactadherin is a popular tool to project cell-targeting proteins on the outer face of the EV membranes [44]. This approach was employed to display an antibody fragment (scFV) to target cancer cells and to present the tumor antigens, OVA and HER2, as cancer vaccines [45]. Similarly, the peptide RPPGSPFR, derived from bradykinin, was used to bind to EVs and other highly curved lipid nanovesicles via lipid chain insertion [46,47]. Collectively, these studies established the usefulness of molecular scaffolds in engineering EVs for use as targeted therapeutic vehicles. Native EVs exhibit broad heterogeneity as a result of different tissue origins, specific surface modifications, and physicochemical properties, all of which may impact their suitability for further engineering of molecular functions for the targeting and delivery of therapeutic agents. EVs derived from a single cell type, however, may alleviate this problem, while genetic manipulation of the host cell may serve to introduce a defined molecular scaffold that presents specific targeting moieties on the external face of the EV and a therapeutic biologic in the lumen.

In this study, we assess the impacts of incorporating transmembrane scaffolds, including CD9, CD63, CD81, and VSVG, on specific physical, molecular, and functional properties of the engineered EVs. Although membrane protein scaffolds have been successfully used to engineer EVs, we know little about the effects of these modifications on different EV subtypes, including potential changes in their diameters, the efficiency of their integration, and altered expression of surface markers. Herein, we employed a newly developed, high-throughput on-chip assay in a comparative study of specific physicochemical properties of engineered EVs at the level of individual vesicles. Our results show that each molecular scaffold exhibits quantitative differences in the efficiencies of their integration into different subtypes of EVs, in the expression levels of specific membrane protein surface markers, and in the uptake of engineered EVs by recipient cells. These findings are important as alterations in the physicochemical and functional properties of engineered EVs may impact their potential for applications in nanomedicine.

## 2. Materials and Methods

### 2.1. Cells and Reagents

Human embryonic kidney cells (293T) were purchased from Alstem (Richmond, CA, USA). High glucose DMEM, Opti-MEM medium, and fetal bovine serum were purchased from ThermoFisher Scientific (Waltham, MA, USA). Chemically-defined and serum-free UltraCulture medium was purchased from Lonza (Portsmouth, NH, USA). The transfection reagent, polyethyleneimine (PEI), was purchased from Sigma-Aldrich (St. Louis, MO, USA). Lipofectamine2000 was purchased from Invitrogen, and FuGene6 were sourced from Promega (Madison, WI, USA). Nuclear staining solution Hoechst 33342 was purchased from ThermoFisher Scientific (Fremont, CA, USA). The EV precipitation solution (ExoQuick-TC) was obtained from System Biosciences (Palo Alto, CA, USA).

### 2.2. Vectors and Fusion Genes

Scaffolds, including human tetraspanins (CD9, CD63, and CD81) and viral VSVG, were fused with copepod GFP at the C-terminus of all scaffolds, as reported previously [39,41]. All expression vectors were constructed similarly. The coding sequences for the exosome-targeting scaffold and GFP fusion were placed after the cytomegalovirus promoter (CMV) but followed by the polyadenylation signal sequences (PolyA). All final constructs were verified by double-stranded DNA sequencing to ensure fidelity.

### 2.3. Cell Culture and Transfection

293T cells were cultured in high-glucose DMEM supplemented with 10% fetal bovine serum, 2 mM GlutMax, and 100 U/mL penicillin-streptomycin. Unless otherwise stated, all transfections were performed in six-well culture plates. Transfection conditions were optimized so that over 80% of cells will be effectively transfected, as judged by GFP-positivity. Typically, cells grown at 40~60% density were transfected by adding plasmid DNA (1~2 μg/well) mixed with transfection reagents (Lipofectamine 2000, FuGene6, or PEI), as previously reported [48].

### 2.4. Isolation of EVs

Surface modification and subsequent preparation of EVs from transfected cells were performed as previously reported [42]. Briefly, 293T cells grown on 15-cm plates (70~80% confluence) were transfected with PEI reagent. 24 h following transfection, cells were switched to a serum-free UltraCulture medium to produce modified EVs. After an additional 48 h of incubation, the conditioned medium was collected, centrifuged, and filtered through a 0.22 μm filter to remove larger EVs (>220 μm). The resulting filtered medium was subsequently mixed with ExoQuick-TC and incubated at 4 °C overnight. The EVs were precipitated after a second round of centrifugation at 3000× *g* for 60 min. The pelleted EVs were gently suspended in a phosphate buffer for further analysis or stored at −80 °C for future use.

### 2.5. Live Cell Microscopy

Cultured cells or fluorescently labeled EVs were monitored by using either an Olympus CKX53 culture microscope or a Leica TCS SP8 confocal microscope. To demonstrate the subcellular localization of GFP reporters, we recorded fluorescence and transmitted light images from the same field. Image adjustments such as brightness and contrast were applied to the entire image frame using dedicated instrument software.

### 2.6. On-Chip Capture and Characterization of EVs

Antibody-based capturing and analysis of single EVs were performed using the ExoView^®^ Tetraspanin Kits (NanoView Biosciences; Brighton, MA, USA) according to the manufacturer’s instructions on the ExoView^®^ R100 platform (NanoView Biosciences), a single particle interferometric reflectance imaging sensing (SP-IRIS) platform coupled with fluorescence detection. Briefly, isolated EVs were first diluted in PBS (~100-fold dilution), and an equal volume of EVs was further diluted in PBS to a final concentration of 0.006 μg/μL. Chips coated with anti-CD9/CD63/CD81 antibodies and murine non-specific control IgG antibodies were used for EV capturing. Fluorescent CF647-anti-CD63 and CF555-anti-CD81 antibodies were used to detect CD63 and CD81 expression on the EV surface. EV sizes were measured by the SP-IRIS mode on ExoView^®^ R100, and the EV surface protein profile (CD63 and CD81) was measured by the fluorescence mode. For all the fluorescence images, surface CD63 and CD81 detected by the fluorescent antibodies and exogenous scaffolds fused to GFP (CD9/CD63/CD81/VSVG) were pseudocolored as red, green, and blue, respectively. The fluorescence thresholds were set to 300 a.u., 300 a.u., and 600 a.u. for the red, green, and blue channels, respectively, throughout each experiment.

### 2.7. Fluorescein-Labeling of EVs

EVs isolated from the conditioned medium were labeled by the ExoGlowTM-RNA EV labeling kit (SBI, Palo Alto, CA, USA) according to the manufacturer’s instructions for flow cytometry analysis. The kit contained reagents specific to EV mRNAs. We labeled both engineered and non-engineered control EVs using this kit and conducted subsequent cellular uptake experiments on fluorescein-labeled EVs.

### 2.8. Cellular Uptake Assay

EV uptake assays were performed as previously reported [48]. Briefly, recipient 293T cells were seeded and incubated in an FBS-supplemented medium overnight for attachment before being exchanged to an FBS-free UltraCulture medium containing fluorescein-labeled EVs to allow cellular uptake to occur. The uptake process of fluorescence-labeled EVs was monitored using fluorescence microscopy. At the indicated time points, we recorded fluorescence and transmitted light images of the preparations.

### 2.9. Flow Cytometry Analysis

Recipient 293T cells treated with increased amounts (0, 3.12, 6.26, 12.5, 25, 50, and 100 ug/mL) of EVs for 24 h were trypsinized, washed, and re-suspended in phosphate buffer. The amount of fluorescein isothiocyanate (FITC)-labeled EV endocytosed by recipient cells was quantified using flow cytometry in an Accuri C6 Plus instrument (BD Biosciences), as described previously [49]. Control cells (not treated with EVs) were also included in the study to determine the background fluorescence level. The fluorescence intensity data from these studies were analyzed using the CFlow Plus software (BD Biosciences).

### 2.10. Data Analysis and Statistics

Data on single EVs were acquired by the ExoScan Software connected to the ExoView^®^ R100 platform. Fluorescence images of the chips and analysis of the integration efficiency of different scaffolds, expression levels of EV surface markers, and sizes of individual vesicles were performed using the NanoViewer software. Statistical analysis of these data was performed using Python 3.8.3. A *p*-value (<0.05) using a Student’s *t*-test was considered statistically significant.

## 3. Results

### 3.1. Engineering Strategy and Experimental Design

**Figure 1:**
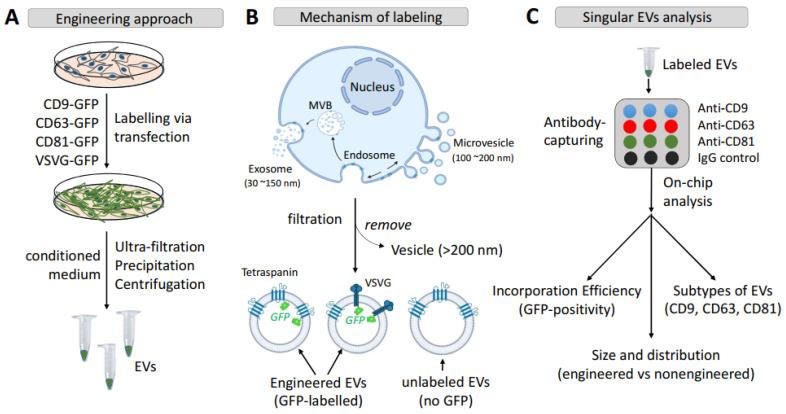
Strategy and molecular mechanisms underlying surface engineering of EVs via transmembrane scaffolds. (**A**) The workflow illustrates the engineering of EVs using either endogenous tetraspanins (CD9, CD63, and CD81) or exogenous vesicular stomatitis virus envelope glycoprotein (VSVG). (**B**) The molecular mechanism underlying the genetic labeling of EVs and membrane topology of the engineering scaffolds. (**C**) Methods used by on-chip analysis for characterization of scaffold-modified vs. non-modified EVs. Abbreviations: vesicular stomatitis virus envelope glycoprotein, VSVG; small extracellular vesicles, EVs; multi-vesicular bodies, MVBs.

Membrane-embedded protein scaffolds are widely used in the engineering of EVs for cell-targeting and loading therapeutic proteins/enzymes [41,50]. Membrane anchoring scaffolds are usually based on integral transmembrane proteins and include tetraspanins, viral envelope glycoproteins [41,50], and membrane-associated proteins such as those with the C1C2 domain [44]. In this study, we focus on transmembrane scaffolds as they provide flexibility in engineering the inner and outer surfaces of EVs [50] for both cell-targeting and drug-loading functions. In particular, we engineered EVs using the intrinsic exosome proteins CD9, CD63, and CD81 (tetraspanins), and the exogenous viral envelope glycoprotein, VSVG. The goal of the study was to investigate whether different molecular scaffolds impact the efficiency of incorporation in the EV membrane, and the potential alteration of physicochemical and biological properties of engineered EVs, including their diameter and molecular profiles. To achieve this goal, we used reporter constructs tagged with GFP previously described by our groups for real-time analysis of engineered secreted EVs (Figure 1A). The GFP molecule was tagged to the C-terminus of each type of protein scaffold to locate the probe on the luminal side of the EV membrane (Figure 1B). This approach was adopted to minimize potential interferences with the membrane targeting and anchoring abilities of the scaffolds. Following the isolation of engineered EVs from conditioned medium, we performed a high-throughput analysis of individual EVs using ExoView^®^ to determine: (1) the incorporation efficiency; (2) evidence of subtypes in the population of engineered EVs; and (3) potential alteration of the size and surface markers (Figure 1C).

### 3.2. Transmembrane Scaffolds Incorporate into Biogenic Sites of EVs in Living Human Cells

**Figure 2:**
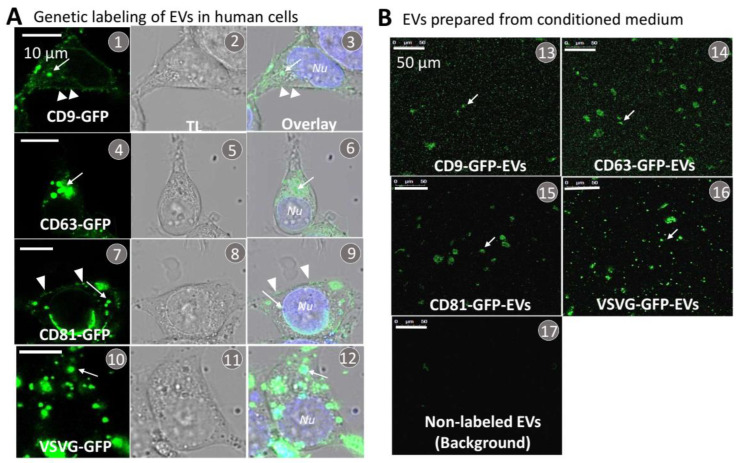
Transfection, expression, and isolation of genetically labeled EVs from conditioned medium of cultured 293T cells. (**A**) Cultured human 293T cells were transfected with each of the transmembrane scaffolds tagged with a GFP reporter, including CD9-GFP (**A1**–**A3**), CD63-GFP (**A4**–**A6**), CD81-GFP (**A7**–**A9**), and VSVG-GFP (**A10**–**A12**). At 48 h following the transfection, both fluorescence and transmitted light images were recorded. Arrows (→) indicate the incorporation of GFP-tagged scaffolds into the endocytic compartment of the endosome and/or MVB, while arrowheads (>) indicate focal incorporation into the plasma membrane. (**B**) EVs were prepared from the conditioned medium collected from the transfected cells, and confocal images were taken at the same dilution (0.1 μg/μL, protein of EVs) for all samples (**B13**–**B16**). Images were also recorded using unlabeled control samples to quantify background noise (**B17**). Arrows indicate individual/clustered EVs. Scale bar, 50 μm.

Genetic fusions of GFP with the identified transmembrane scaffolds were developed to enable real-time monitoring and imaging of their incorporation and subsequent loading into the EV membranes within live human cells. We confirmed these scaffolds were correctly targeted to EVs by first transfecting each of the GFP-fusion constructs in 293T cells and then imaging the GFP fluorescence to quantify their expression levels and integration in EV membranes. Confocal fluorescence microscopy images of GFP showed that all of the transmembrane scaffolds were able to localize to some areas of the plasma membrane (arrowheads, Figure 2(A1,A3,A7,A9)), endocytic compartments (arrows, Figure 2(A1–A12)), and sometimes the periphery of nucleus (Figure 2(A9)). Further analysis of GFP image data supports the view that all of the transmembrane scaffolds (CD9, CD63, CD81, and VSVG) were successfully targeted and incorporated in EV membranes. These findings are consistent with previous reports showing tetraspanins (CD9/CD63/CD81) and VSVG incorporate into the plasma membrane and/or endocytic compartments such as multi-vesicular bodies (MVBs) and are secreted as EVs to the extracellular environment [51,52,53]. While all tested GFP fusions localized to the plasma membrane and endocytic compartments, we found CD63 and VSVG were more likely to concentrate in the endocytic compartment, as seen by the larger number of GFP puncta in the cytosol (Figure 2(A4–A6,A10–A12)). In contrast, CD9 and C81 showed greater GFP fluorescence signals at the plasma membrane and less in endocytic compartments (Figure 2(A1–A3,A7–A9)). These differences in membrane distribution could suggest specific types of the transmembrane scaffold may result in the formation of different types of EVs, i.e., not just exosomes (Figure 1B). Please note that the GFP granules that appear in the cytosol are most likely MVBs rather than pre-secreted individual exosomes. Because MVBs may contain different numbers of exosomes, GFP granules tend to have different sizes (Figure 2(A4,A10)).

Next, we investigated whether purified EVs secreted from transfected 293T cells incorporated their respective GFP-tagged scaffolds. Briefly, EVs were purified from conditioned medium by using a combination of ultrafiltration, chemical precipitation, and centrifugation methods, as previously reported [42]. A small amount of the purified EV preparation was smeared on a microscope cover slip for subsequent fluorescence imaging and image analysis. Correct integration of each scaffold in the EV membrane was indicated by strong GFP signals (Figure 2(B13–B16)) whereas control EVs did not generate any significant fluorescence signal (Figure 2(B17)).

### 3.3. Each Transmembrane Scaffold Preferentially Incorporates into Different Subtypes of EVs with Various Efficiency

**Figure 3:**
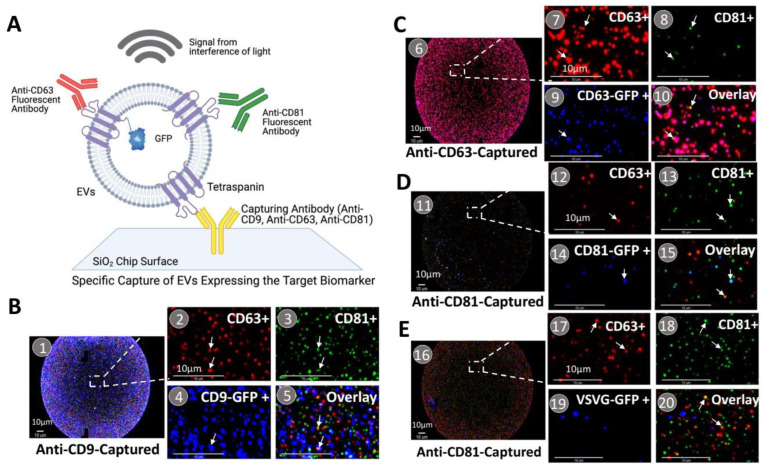
On-chip analysis of antibody-captured EVs. (**A**) A schematic representation of on-chip analysis of genetically labeled EVs. The size of EVs was measured and calculated using signals from the interference of light (interferometry). Surface marker profiles of EVs were specifically detected by fluorescein-labeled monoclonal antibodies. (**B**–**E**) Molecular profiling of various EV subtypes and subpopulations of scaffold-modified EVs. (**B1**–**B5**) CD9-GFP-modified EVs captured by anti-CD9 antibodies. (**C6**–**C10**) CD63-GFP-modified EVs captured by anti-CD63 antibodies. (**D11**–**D15**) CD81-GFP-modified EVs captured by anti-CD81 antibodies. (**E16**–**E20**) VSVG-GFP-modified EVs captured by anti-CD81 antibodies. A portion of the spots (**B1**,**C6**,**D11**,**E16**) was magnified to show the individual EVs and expression/co-expression of the surface markers, CD9/CD63/CD81/scaffold-GFP. Arrows indicate co-expression of surface markers on individual vesicles; Scale bar, 10 μm.

The ExoView system provides a powerful method to characterize individual engineered EVs (Figure 3A). Individual EVs are bonded to a layered silicon substrate chip coated with an array of capture antibodies and subsequently characterized using a combination of single-particle interferometric imaging and immunofluorescence imaging modalities. In this way, the system enables one to profile different EV subpopulations and determine the successful incorporation of each transmembrane scaffold into individual EVs. In our studies, we captured EVs at different sites on the chip using surface-attached monoclonal antibodies directed against CD9, CD63, and CD81. Bonded EVs were then fluorescently stained using the following antibody conjugates: CF647-anti-CD63 (red) and CF555-anti-CD81 (green) to profile these surface markers. The workflow used to characterize EV populations in a sample was as follows: first identify engineered EVs on the chip by imaging GFP fluorescence for each subpopulation of EV on the chip (CD9^+^, CD63^+^, or CD81^+^; Figure 3B–E). For example, Figure 3(B1) shows a GFP-fluorescence image of CD9-GFP-modified EVs captured at a site with surface-attached anti-CD9 antibodies (GFP emitting is pseudotyped with blue color). High-resolution multispectral analysis of fluorescence images of immunostained EVs on the chip (Figure 3(B2–B5)) is then used to identify individual EVs with a specific surface marker profile. For example, GFP harboring EVs that emit red emissions are CD63 positive, those with green emissions are CD81 positive, and those with blue emissions are GFP positive. Overlay images of the three colors are then used to quantify the amounts of each biomarker protein on the membrane surface of the EVs. This approach was used to study the molecular profiles of EVs harboring the different molecular scaffolds, namely CD63-GFP, CD81-GFP, and VSVG-GFP, shown respectively in Figure 3(C6–C10,D11–D15,E16–E20).

**Figure 4:**
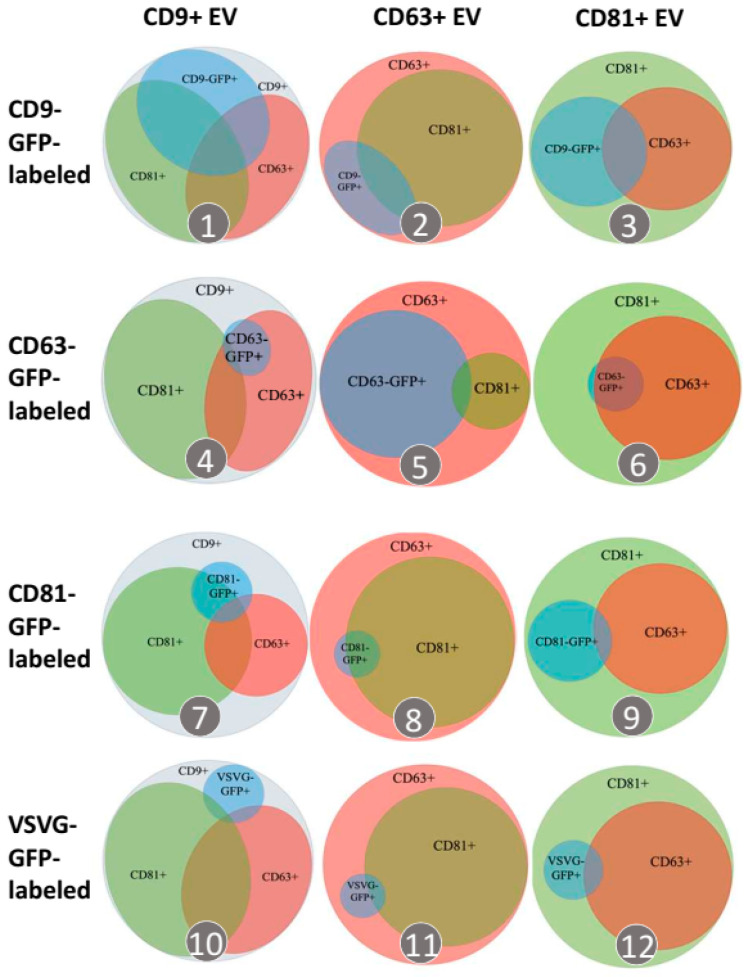
Transmembrane scaffolds incorporate into distinct EV subtypes. Venn diagrams summarize percentages of labeled EVs in each distinctive subpopulation of EVs. EVs captured by anti-CD9, anti-CD63, or anti-CD81 antibodies are characterized as CD81^+^ (green outer circle), CD63^+^ (red outer circle), or CD9^+^ (grey outer circle) subpopulations, respectively (columns). The percentage of EVs modified by different transmembrane scaffolds, including CD9-GFP-, CD63-GFP-, CD81-GFP, or VSVG-GFP, is represented by the blue inner circle. The surface marker profiles of CD63 and CD81 determined by fluorescein-labeled anti-CD63 and anti-CD81 antibodies were represented by the smaller red and green inner circles/ellipses, respectively. The size of all circles and ellipses was proportional to their percentages.

Next, we characterized GFP-positive/negative EVs in terms of the molecular profile of surface markers using Venn diagrams (Figure 4(1–12)). The results show that each tetraspanin can be incorporated into its respective subgroup. More specifically, CD9-GFP preferentially modifies CD9^+^ EVs (47.2%) as compared to CD63^+^ (19.7%) and CD81^+^ (32.4%) EVs. Similarly, CD63-GFP and CD81-GFP predominantly modify CD63^+^ (58.7%) and CD81^+^ (16.6%) EVs, respectively. In contrast, viral VSVG-GFP scaffolds have low efficacy in incorporating into all three subgroups of EVs (8.3% in CD9^+^, 4.7% in CD63^+^, and 8.5% in CD81^+^ EVs) (Table 1 in Figure 4).

### 3.4. Surface Engineering Using Transmembrane Scaffolds Leads to an Increase in EV Size

**Figure 5:**
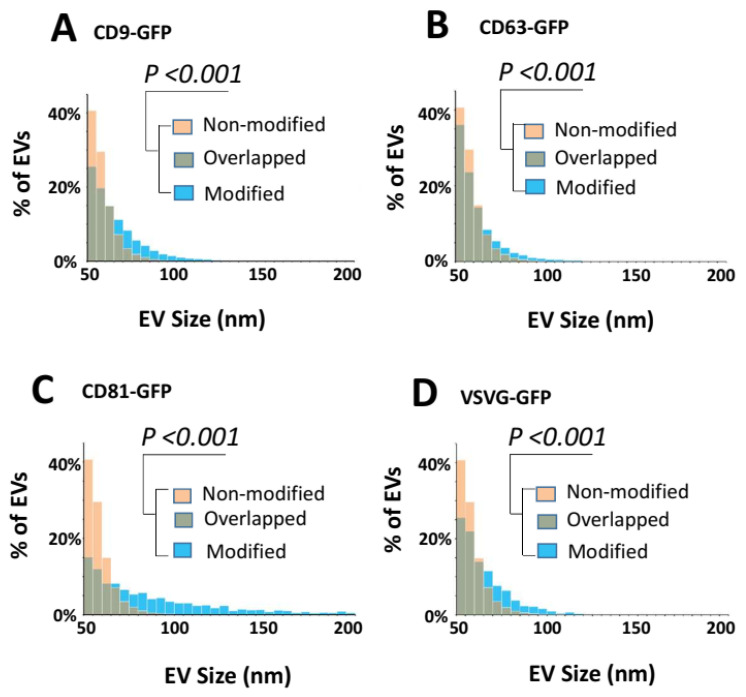
Transmembrane scaffolds increase the size of EVs. (**A**–**D**) EVs prepared from the conditioned medium were subjected to on-chip analysis. The vesicle size distributions were graphed for CD9-GFP-modified vs. non-modified EVs (**A**), CD63-GFP-modified vs. non-modified EVs (**B**), CD81-GFP-modified vs. non-modified EVs (**C**), or VSVG-GFP-modified EVs vs. non-modified EVs (**D**). Yellow and blue bars represent non-modified control and transmembrane scaffold-modified EVs, respectively, while green bars represent the overlay portion. The counts of EV numbers are normalized to the percentages of total vesicle numbers analyzed and graphed. The size differences between the modified and non-modified groups are statistically significant with a *p*-value < 0.001 (Student’s *t*-test).

Next, we examined the impact that different transmembrane scaffolds may have on the diameter of EVs. Taking advantage of the high-throughput power of single EV analysis via SP-IRIS (single particle interferometric reflectance image sensor), we measured the diameters of 64,861 individual EVs captured by surface-bound anti-CD9/CD63/CD81 antibodies (Supplemental Table S1). As shown in Figure 5A–D, the integration of transmembrane scaffolds in EVs led to significant increases in the diameter of all engineered EVs. The average size of unmodified EVs was 58.4 ± 8.07 nm, while the mean diameter of modified EVs was all larger than 60 nm. More specifically, CD81-GFP led to the largest increase (86.02 nm ± 34.18), followed by CD9-GFP (66.13 nm ± 16.38), VSVG-GFP (65.42 nm ± 15.62), and CD63-GFP (62.07 nm ± 14.20), respectively (Table 2). While the average percentage of unmodified EVs having a diameter ≥ 60 nm was <29%, the same measure for EVs bearing the CD81-GFP scaffold was 72.9%, 54.7% for CD9-GFP, 52.5% for VSVG-GFP, and 40.3% for CD63-GFP (Supplemental Table S2). Thus, if the diameter of the EVs was found to negatively impact their physiological function, then one would likely select EVs harboring the CD63-GFP scaffold, followed by VSVG-GFP, CD9-GFP, and CD81-GFP.

### 3.5. CD63 Scaffold Decreases the Expression Levels of CD81 Markers on Modified EVs

**Figure 6:**
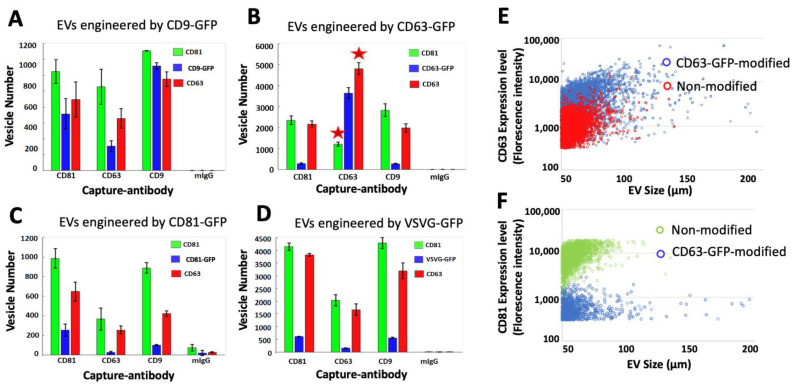
Effects of the engineering scaffolds on the expression of EV surface markers. (**A**–**D**) EVs captured on-chip by monoclonal antibodies to CD9/CD63/CD81 were further characterized for their expression of the GFP-tagged engineering scaffolds (blue) as well as CD63 and CD81, which are detected by the fluorescein CF647-labeled anti-CD63 antibody (red) and the CF555-labeled anti-CD81 antibody (green). The number of EVs that express these markers was analyzed and graphed. (**E**,**F**) Scatter plots comparing the expression levels of surface markers in CD63-GFP-modified vs. non-modified EVs. Blue dots represent CD63-GFP-EVs and red dots represent non-labeled EVs (**E**); Blue dots represent CD63-GFP-EVs and green dots represent non-labeled EVs (**F**).

Next, we examined differences in the expression levels of specific tetraspanin membrane proteins such as CD63 and CD81 in EVs bearing different transmembrane scaffolds [54]. This quantitative study revealed that the CD63-GFP scaffold decreased the amount of CD81 in EVs (Figure 6B). whereas no change in the expression level of CD81 was evident when other transmembrane scaffolds such as CD9, CD81, or VSVG were used (Figure 6A,C,D). Specifically, CD81 was lowered by ~50% in EVs captured by anti-CD63 antibodies, and this decrease in the levels of CD81 was accompanied by an increase in CD63 by ~40% (indicated by stars in Figure 6B). In contrast, EVs captured by other surface marker antibodies such as CD81 and CD9 exhibited higher levels of CD81 expression than CD63. These results suggest that CD63 modification may cause a decrease in the levels of CD81^+^ in CD63-positive EVs.

Further confirmation that CD63-GFP incorporation in EVs decreases the expression of CD81 coupled with an increased CD63 expression came from an analysis of fluorescence intensity measurements of CD63 and CD81 in CD63-GFP and non-labeled control EVs. A plot of CD63 fluorescence intensity vs. average diameter reveals that individual EVs bearing CD63-GFP have higher CD63 fluorescence compared to non-labeled EVs (Figure 6E, blue dots vs. red dots), indicating an increase in the expression of CD63. In contrast, when we plot the CD81 fluorescence intensity, the two groups show an opposite distribution pattern: the CD63-GFP-modified EVs have a lower CD81 expression level compared to the non-labeled EVs (Figure 6F, blue dots vs. green dots). This analysis confirms that engineering EVs with CD63-GFP can cause a decrease in the levels of expression of CD81 but an increase in the levels of CD63 expression.

### 3.6. The VSVG Scaffold Increases the Cellular Uptake of EVs

**Figure 7:**
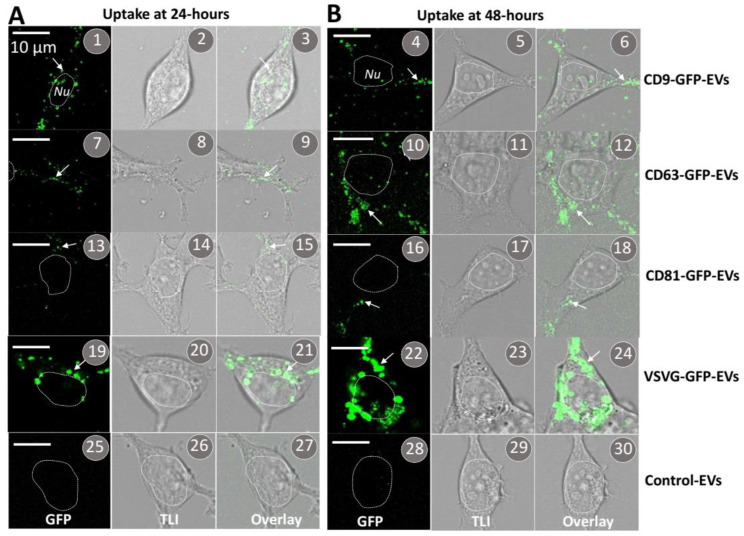
Cellular uptake of EVs modified by different transmembrane scaffolds. EVs prepared from the conditioned medium of transfected cells were added to the medium (30 μg EV proteins/mL) of the cultured 293T cells. Confocal images were recorded at 24 h (**A**) and 48 h (**B**) after the addition of CD9-GFP-modified EVs (**1**–**6**), CD63-GFP-modified EVs (**7**–**12**), CD81-GFP-modified EVs (**13**–**18**), VSVG-GFP-modified EVs (**19**–**24**), or non-modified controls (**25**–**30**). Arrows indicate internalized EVs. Scale bar, 10 μm.

Next, we determined whether scaffold-modified EVs are capable of being endocytosed by recipient cells. Purified EVs genetically labeled with GFP-tagged scaffolds were added to the culture medium of 293T cells for either 24- or 48-h, and after washing the cells, they were imaged by confocal microscopy (Figure 7). Confocal fluorescence images showed GFP granules in cells with EVs bearing GFP fusions of CD9/CD63/CD81/VSVG-GFP (Figure 7; arrows). Consistent with previous reports [41], analysis of confocal fluorescence images seen at the 24- and 48-h time points showed EVs harboring the VSVG scaffold were taken up to a greater degree than EVs modified with tetraspanin scaffolds (Figure 7(1–18) vs. Figure 7(19–24)). Since EVs bearing each type of transmembrane scaffold were taken up by recipient cells, we conclude the integration of the scaffold protein and its GFP cargo to the EV membrane does not impact their ability to undergo internalization. Moreover, we envisage opportunities to fuse other protein cargo to the scaffold that may function as a therapeutic, for example, a functional enzyme for the treatment of a lysosomal storage disorder.

**Figure 8:**
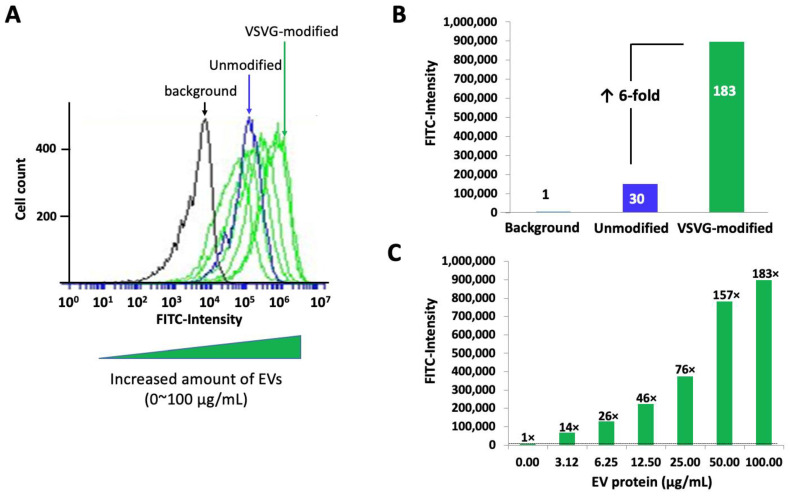
VSVG-GFP-modified EVs dramatically increase the cellular uptake by recipient cells. EVs prepared from the conditioned medium of either VSVG-GFP-modified cells or non-modified controls were chemically stained by Exoflow before being added to the culture medium of human 293T cells. The cells were treated for 24 h with increased concentrations of EVs (3.12–100 EV protein/mL), then washed, collected, and subjected to flow cytometry analysis as described in Materials and Methods. The amount of EV uptake was analyzed and graphed with CFlow Plus software. (**A**) A dose-response curve of VSVG-modified EVs compared to the control. The cellular uptake of VSVG-modified EVs (green) dramatically increased (shift to the right) compared to the non-modified control (blue) at the same EV concentration (100 μg of protein/mL) and the background (black). (**B**) A bar-graph representation of the relative uptake efficiency of VSVG-GFP-modified (green) vs. non-modified control (blue) vs. the background (black). (**C**) A bar-graph representation of increasing cellular uptake of VSVG-modified EVs in a dose range of 0–100 μg EV protein/mL.

Unlike the tetraspanin-based transmembrane scaffolds, VSVG is a single-span transmembrane glycoprotein whose large ectodomain projects to the external environment of the EV. This ectodomain binds specifically to the low-density lipoprotein receptors (LDLRs) found on the surface of almost all cell types [55]. Interactions between VSVG and the receptor are known to lead to receptor-mediated endocytosis [56]. Armed with this knowledge, we propose the ectodomain of VSVG on engineered EVs is recognized by LDLRs on 293T cells, triggering their endocytosis, which may account for the increased levels of cellular uptake of EVs bearing VSVG compared to tetraspanins. To further validate that the VSVG scaffold increases the cellular uptake of modified EVs by its ectodomain, we conducted a quantitative flow cytometry analysis of cells treated with either VSVG-modified EVs, control (non-modified) EVs, or no EVs. After fluorescein labeling with the ExoGlow^TM^ reagent, identical quantities (100 μg/mL) of VSVG-modified and non-modified control EVs were added to the culture medium of 293T cells and allowed to incubate for 24 h. Next, 293T cells were subjected to flow cytometry analysis to record the FITC intensity in individual cells. We found that the intensity of FITC fluorescence in control EVs (Figure 8A,B, blue curve + bar) was 30-times higher than the FITC intensity in cells that were not treated with EVs (Figure 8A,B, black curve + bar). In comparison, the FITC intensity in cells treated with VSVG-modified EVs was 180 times higher than the GFP-channel intensity in untreated cells and 6-fold higher than the uptake of control EVs. Thus, EVs integrating VSVG exhibit enhanced endocytosis-mediated uptake. Next, we conducted a dose-response study to determine the effective range of VSVG-modified EVs for intracellular delivery of fused molecular cargo. From the plot of the amount of VSVG-modified EVs taken up by cells versus the amount of EVs added to the medium (3.12, 6.25, 12.5, 25.0, and 50.0 μg/mL), it is evident that saturation occurs at an EV level of ~50 μg/mL (Figure 8C).

## 4. Discussion

The long-term goal of these studies is to optimize the design and engineering of EVs as living vehicles for the delivery of therapeutic biologics [44,57,58,59]. In particular, we aimed to characterize the properties of EVs harboring different molecular scaffolds that can be engineered for specific tissue targeting and uptake by recipient cells. In engineering EVs for clinical applications, it will be essential to establish how specific molecular scaffolds impact the physicochemical and biological phenotypes of modified EVs. Ultimately, this knowledge will help narrow the types of transmembrane scaffolds that are suitable for EV-based therapies [60,61]. In the present study, we employed state-of-the-art high-throughput, on-chip assays to examine how different scaffolds affect the engineering efficiency, physical properties, and biological responses of engineered EVs [39,41,60,62,63]. We found that the endogenous tetraspanin scaffolds (CD9, CD63, and CD81) were incorporated into distinct subpopulations of EVs, whereas VSVG was found equally in all EV subtypes. We also established that EVs bearing molecular scaffolds increased their average diameter compared to unmodified EVs. Although we do not know the exact mechanisms accounting for the increased size of the engineered EVs, we speculate the following three possibilities: (1) loading of GFP cargo into the lumen of engineered EVs may account for increased overall cargo loading, thus an increase in the size of engineered EVs; (2) incorporation of transmembrane scaffolds may alter the expression of endogenous surface markers; for example, the use of CD63-GFP scaffolds resulted in a decrease in the levels of CD81 (Figure 6). The change in membrane protein profile of engineered EVs may also lead to a change in EV sizes; (3) because all transmembrane scaffolds are glycoproteins, the incorporation of additional glycoproteins could increase the overall size of engineered EVs due to two mechanisms: the large and projecting ectodomain of VSVG and more sugars brought onto the outer surface by scaffold glycoproteins. We also showed EVs bearing the CD63 scaffold exhibit different expression levels of validated EV membrane biomarkers, specifically CD63 and CD81. We further demonstrated that EVs harboring VSVG were taken up by recipient cells to a greater degree (×3) compared to EVs bearing tetraspanins. Significantly, the exodomain of VSVG is known to interact with the LDL receptors on the outer membrane of most cells, where it is internalized by endocytosis. As such, this class of engineered EV may prove useful in delivering an internal cargo of functional enzymes to cells associated with LSDs [60]. In summary, our study has yielded important insights into the design and engineering of EVs with transmembrane scaffolds that may be important in the development of EVs for clinical applications.

Our study has painted a detailed picture of EV biogenesis by monitoring the subcellular localization and, finally, incorporation into different subtypes of EVs via different transmembrane scaffolds. It is well established that tetraspanins and VSVG initially enter the ER membrane after their synthesis but are quickly funneled into the plasma membrane (PM) via the route of the ER-Golgi-secretory vesicles [64,65]. After arriving at the PM, these transmembrane scaffolds may be differently sorted into distinctive microdomains, including tetraspanin-enriched microdomains or lipid rafts [66]. Existing evidence supports the hypothesis that CD9 and CD81 tend to interact with each other in certain domains that can bud off from the PM to become EVs [8,67,68]. In contrast, CD63 and VSVG may accumulate in other areas, where they undergo inward budding to form endosomes and then become intraluminal vesicles (pre-secreted exosomes) within MVBs [8]. This conjecture is supported by our image evidence that CD9 and CD81 are more enriched in the PM while CD63 and VSVG are localized mainly in endocytic compartments (Figure 2). Of note, CD9 is more often co-expressed on the same EVs as CD81 than with CD63 or VSVG, supporting the notion that CD9 and CD81 are more closely associated but divergent from CD63 and VSVG (Figure 6). Furthermore, the average size of EVs modified by CD9-GFP or CD81-GFP is slightly larger than that of those modified by CD63 or VSVG, suggesting different engineering effects on PM-derived and endosome-derived EVs (Table 2). Although molecular interactions among different tetraspanins are not mutually exclusive, overexpression of CD63-GFP appears to decrease the levels of CD81^+^ EVs (Figure 6), supporting a stronger CD63-CD63 rather than CD63-CD81 association. Additionally, VSVG has a low co-localization rate with the tetraspanins (Table 1), indicating independent self-trimerization [69,70] rather than through interactions with tetraspanins.

The findings of our study may have important implications for the development of EV-based therapeutics using these transmembrane scaffolds. First, we find that both CD63 and VSVG preferentially modify endosome-derived EVs, which are commonly defined as exosomes. It has been established that exosomes are enriched with antigen-presenting molecules such as major histocompatibility complex I (MHC-1) and MHC-2 molecules [71,72]. Therefore, CD63 and VSVG may be more suitable for certain applications, such as the development of exosome-based nanovaccines to improve immune response [73,74,75]. VSVG has been used to present cancer and other antigens to elicit robust immune responses in animal models [62]. CD63 has also been proven to be a useful scaffold for the engineering of exosome-based vaccines [50,76]. On the other hand, CD9 and CD81 may be more suitable for targeted drug delivery because their modified EVs tend to become larger, allowing more drugs to be loaded into EVs [77]. Interestingly, CD9 has been successfully engineered to load cytosolic proteins or enzymes into EVs [78], while CD81 has also been mutated to gain the cancer-targeting ability for potential anticancer therapy [40].

Second, we found that individual scaffolds can only modify a small fraction of EVs, less than 50% even for the dominant subtypes of EVs (Table 1). Therefore, existing preparation protocols such as ultrafiltration, centrifugation, and even immunoaffinity capture may not be sufficient to isolate the modified EVs; hence, more robust and precise purification protocols must be individually developed. For instance, by adding a small affinity tag, such as 6xHistag, to the engineering scaffolds of CD63/VSVG, one may use nickel-coated magnetic beads to specifically capture the scaffold-modified EVs, therefore obtaining better enrichment of modified EVs. Such a specific purification method is especially important for the VSVG scaffold because VSVG-modified EVs normally lack the common surface markers (CD9, CD63, and CD81).

Third, we find that the VSVG viral scaffold may well preserve its intrinsic functions that are widely exploited, including the generation of VSVG-pseudotyped EVs for enhanced intracellular delivery via its ectodomain [41,42,56,79,80]. By the same principle, one may also use other viral glycoproteins to program EVs for tissue/cell-specific delivery. For example, spike protein from the COVID-19 coronavirus may render a tissue-specificity to epithelial/endothelial cells expressing receptor angiotensin-converting enzyme-2 receptors on their surface [81]. Lastly, we find the EVs may be vulnerable to some phenotypical alterations, including an increase in the size of modified EVs and changes in the levels of certain surface markers like CD63 and CD81 [82]. Although the biological significance of these changes remains to be determined, stringent quality control measures may be required to evaluate any changes in tissue tropics, pharmacological dynamics and kinetics, and biological activities before using them for clinical applications [83].

## 5. Conclusions

We have built up a set of genetic fusion reporters, which enable direct assessment of the engineering efficacy and biological impacts of various transmembrane scaffolds on modified EVs. Molecular imaging and single EV analysis reveal that these transmembrane scaffolds may have different abilities to engineer distinctive subtypes of EVs. Surface modification of EVs via various scaffolds may change physiochemical properties, such as vesicle size, surface protein marker expression, and membrane functioning via scaffold’s ectodomain, of successfully modified EVs. Our findings may have important implications for the genetic engineering of EVs using transmembrane scaffolds.

## Figures and Tables

**Table 1:** Engineering efficiency with different scaffolds.

	CD9^+^ EVs	CD63^+^ EVs	CD81^+^ EVs
CD9-GFP-EVs	47.2% ± 0.8%	19.7% ± 5.1%	32.4% ± 4.0%
CD63-GFP-EVs	6.7% ± 0.1%	58.7% ± 1.5%	7.3% ± 0.5%
CD81-GFP-EVs	12.3% ± 6.3%	4.9% ± 0.6%	16.6% ± 2.0%
VSVG-GFP-EVs	8.3% ± 0.2%	4.7% ± 0.8%	8.5% ± 0.1%

Note: Data is represented in mean ± standard deviation (n = 3).

**Table 2:** Effects on the size of EVs via transmembrane scaffold.

Name of Scaffold	Unmodified EVs (nm, size)	Modified EVs (nm, size)
CD9-GFP	58.40 ± 8.07	66.13 ± 16.38
CD63-GFP		62.07 ± 14.20
CD81-GFP		86.02 ± 34.18
VSVG-GFP		65.42 ± 15.62

## Supplementary Materials

The following supporting information can be downloaded at: https://www.mdpi.com/article/10.3390/pharmaceutics14122564/s1, Table S1: Number of particles captured and measured in each subtype of EVs; Table S2: Increased sizes of EVs via various engineering scaffolds.
