# Supplementary material for: Genetically Engineered Extracellular Vesicles Harboring Transmembrane Scaffolds Exhibit Differences in Their Size, Expression Levels of Specific Surface Markers and Cell-Uptake
Source: Pharmaceutics. 2022 Nov 23;14(12):2564. doi: 10.3390/pharmaceutics14122564 (PMC9783873; doi:10.3390/pharmaceutics14122564)
Supplement: Supplementary file 1 [file pharmaceutics-14-02564-s001.zip › pharmaceutics-2013372-supplementary.pdf]

**Supplemental Table S1. Number of particles captured and measured in each subtype of EVs**

| Name of Scaffold | Unmodified EVs (number of particles) | Modified EVs (number of particles) |
|---|---|---|
| CD9-GFP | 31927 | 22504 |
| CD63-GFP | | 6681 |
| CD81-GFP | | 1825 |
| VSVG-GFP | | 1924 |
| Total No. | 31927 | 32934 |

**Supplemental Table S2. Increased sizes of EVs via various engineering scaffolds**

| Name of Scaffold | Unmodified EVs (% EV > 60 nm) | Modified EVs (% EV > 60 nm) |
|---|---|---|
| CD9-GFP | 29.7 | 54.7 |
| CD63-GFP | | 40.3 |
| CD81-GFP | | 72.9 |
| VSVG-GFP | | 52.5 |
